# Spinal Cord Cells from Pre-metamorphic Stages Differentiate into Neurons and Promote Axon Growth and Regeneration after Transplantation into the Injured Spinal Cord of Non-regenerative *Xenopus laevis* Froglets

**DOI:** 10.3389/fncel.2017.00398

**Published:** 2017-12-13

**Authors:** Emilio E. Méndez-Olivos, Rosana Muñoz, Juan Larraín

**Affiliations:** Center for Aging and Regeneration, Faculty of Biological Sciences, Pontificia Universidad Católica de Chile, Santiago, Chile

**Keywords:** *Xenopus laevis*, spinal cord injuries, transplantation, regeneration, neural stem cells

## Abstract

Mammals are unable to regenerate its spinal cord after a lesion, meanwhile, anuran amphibians are capable of spinal cord regeneration only as larvae, and during metamorphosis, this capability is lost. Sox2/3^+^ cells present in the spinal cord of regenerative larvae are required for spinal cord regeneration. Here we evaluate the effect of the transplantation of spinal cord cells from regenerative larvae into the resected spinal cord of non-regenerative stages (NR-stage). Donor cells were able to survive up to 60 days after transplantation in the injury zone. During the first 3-weeks, transplanted cells organize in neural tube-like structures formed by Sox2/3^+^ cells. This was not observed when donor cells come from non-regenerative froglets. Mature neurons expressing NeuN and Neurofilament-H were detected in the grafted tissue 4 weeks after transplantation concomitantly with the appearance of axons derived from the donor cells growing into the host spinal cord, suggesting that Sox2/3^+^ cells behave as neural stem progenitor cells. We also found that cells from regenerative animals provide a permissive environment that promotes growth and regeneration of axons coming from the host. These results suggest that Sox2/3 cells present in the spinal cord of regenerative stage (R-stage) larvae are most probably neural stem progenitor cells that are able to survive, proliferate, self-organize and differentiate into neurons in the environment of the non-regenerative host. In addition, we have established an experimental paradigm to study the biology of neural stem progenitor cells in spinal cord regeneration.

## Introduction

Worldwide an estimate of 3 million people live with Spinal Cord Injury (SCI) and approximately 180,000 new cases happen every year with severe implications for their quality of life (Lee et al., 2014). Paraplegia and quadriplegia are a consequence of the inefficient regenerative ability of the mammalian central nervous system (CNS). Different mechanisms involved in this lack of regeneration have been described, on one hand the presence of a non-permissive environment formed by a glial scar containing reactive glia, fibroblasts and meningeal cells, myelin-associated proteins and extracellular matrix components that all together inhibit axonal growth (Burda and Sofroniew, 2014) and also the lack of intrinsic regenerative programs in most adult neurons (He and Jin, 2016). In addition, although neural stem and progenitor cells (NSPC) are present in encephalic niches and physiological neurogenesis has been described (Kriegstein and Alvarez-Buylla, 2009), there is no formation of new neurons in the spinal cord in response to injury (Meletis et al., 2008; Sabelström et al., 2013). In those cases were NSPC have been detected in the spinal cord, cells that enter proliferation after injury are only fated to differentiate into astrocytes and oligodendrocytes (Horner et al., 2000; Meletis et al., 2008). The proliferation of ependymal cells has been observed in association with spinal cord growth (Alfaro-Cervello et al., 2012). Recently it has been reported that ependymal cells proliferate after injury and form astrocytes and oligodendrocytes that contribute to a glial scar that limits the secondary damage (Meletis et al., 2008; Sabelström et al., 2013), although this evidence seems controversial (Ren et al., 2017).

Non-mammalian vertebrates, including lampreys, teleost fishes, amphibians and lizards (Becker and Becker, 2007; Diaz Quiroz and Echeverri, 2013; Lee-Liu et al., 2013) are able to regenerate their CNS. These species achieve spinal cord recovery through axon regeneration, reconstitution of the ependymal channel and neurogenesis implicating the formation of new neurons from NSPC present on the spinal cord (Tanaka and Ferretti, 2009). Among amphibians urodeles (salamanders, newts) maintain their regenerative capacity throughout their lives, but anurans such as *Xenopus laevis* frogs are able to efficiently regenerate after full spinal cord transection at stages before metamorphosis (Regenerative stages, R-stages) and this ability is lost during metamorphosis and juvenile froglets (non-regenerative stages, NR-stages) are no longer able to recover from SCI (Forehand and Farel, 1982; Beattie et al., 1990; Diaz Quiroz and Echeverri, 2013; Lee-Liu et al., 2013, 2016).

In frogs, motor and sensory information from the spinal cord is mainly integrated at the level of the brainstem (mesencephalon and rhombencephalon), instead of the prosencephalon as in mammals, implicating the absence of corticospinal tracts. Most descending supraspinal neurons are already born at stage 53 (R-stage) and their axonal tracts extend into the spinal cord at different stages during metamorphosis (van Mier and ten Donkelaar, 1984; Sánchez-Camacho et al., 2001, 2002). Anterograde and retrograde labeling experiments carried out in *X. laevis* demonstrated that in R-stage animals supraspinal axons can grow and regenerate through the injury site, but ascending sensorial nerves are unable to regenerate (Beattie et al., 1990; Gibbs and Szaro, 2006; Gibbs et al., 2011; Lee-Liu et al., 2016). On the contrary and in correlation with the absence of functional recovery, reconstitution of the descending supraspinal projections was not detected in NR-stages (Beattie et al., 1990). This failure to regenerate could be explained because of the absence of an intrinsic mechanism for axon regeneration and the presence of a non-permissive environment.

Sox2 is a transcription factor necessary for maintaining self-renewal and pluripotency in most stem cells including NSPC and is also required for cell reprogramming (Avilion et al., 2003; Graham et al., 2003; Ferri et al., 2004; Sarkar and Hochedlinger, 2013). The spinal cords of *X. laevis* regenerative larvae, zebrafish and axolotl are abundant in NSPC expressing Sox2 and these cells are activated in response to SCI and are necessary for tail and spinal cord regeneration (Gaete et al., 2012; Fei et al., 2014; Hui et al., 2015; Muñoz et al., 2015). After injury Sox2/3^+^ cells proliferate, self-organize in neural tube-like structures, and differentiate into neurons (Muñoz et al., 2015). Electron microscopy analysis from injured R-stage animals showed axons growing in close association with ependymal processes in the ablation gap, suggesting the ependymal cells (Sox2/3^+^ cells) provide molecular cues for axon regeneration (Michel and Reier, 1979). Contrary to that, Sox2/3^+^ cells are less abundant in the spinal cord of NR-stages of *X. laevis* (Muñoz et al., 2015) and in adult rodents, interestingly they are poorly activated in response to injury correlating with the absence of efficient regeneration (Hamilton et al., 2009).

Currently, no effective therapy for SCI is available. Cell transplantation strategies have been proposed and tested as a potential therapy to improve neural regeneration. Early phase clinical trials have been performed showing that cell transplantation using different cell types from embryonic and adult tissues (e.g., embryonic stem cells, Schwann cells, oligodendrocytes precursors, olfactory ensheathing cells, NSPC and mesenchyme stem cells) is achievable (Tetzlaff et al., 2011; Li and Lepski, 2013). Despite the intense research in cell transplantation little is known about the mechanisms involved in the ability of transplanted cells to improve repair and functional recovery and more knowledge is necessary to attain significant functional improvement, long-term effects, and optimal safety levels. Possible mechanisms involved in how NSPC can improve regeneration include: promote axon regeneration/sprouting, neuroprotection, modulation of glial scar formation, immunomodulation, neurogenesis and myelin regeneration (Assinck et al., 2017).

The Tuszynski laboratory has carried one of the most successful experiments of cell transplantation in rodents. Spinal cords from rodent embryos containing NSPC were dissociated and embedded in fibrin matrices together with a cocktail of growth factors (GF) and then transplanted into fully transected spinal cords resulting in the formation of neurons that extended large numbers of axons at long distances with the formation of electrophysiological relays and mature synapses with host axons that allowed significant functional recovery (Lu et al., 2012). Moreover, the same strategy promotes regeneration in the largely refractory corticospinal tract especially when the transplanted cells come from the caudal region of the spinal cord (Kadoya et al., 2016). These results support the idea that transplanted NSPC can make new neurons in the injury site and also enhance intrinsic properties of host neurons to overcome the non-permissive environment present in the injured spinal cord (Lu et al., 2012). Although very successful more work needs to be done to allow translational impact, especially because it was reported that the transplanted NSPC can migrate to other areas of the CNS and form ectopic colonies with potential detrimental effects (Steward et al., 2014; Assinck et al., 2017).

In order to understand the mechanism and effects of cell transplantation on improving spinal cord recovery here we have established experimental conditions to allow successful transplantation of dissociated spinal cords isolated from *X. laevis* R-stages into NR-stage froglets in which the spinal cord was previously resected. Using this experimental approach here we showed that transplanted cells from R-stage spinal cords, mainly Sox2/3^+^ cells, can survive and proliferate in the host environment and self-organize in neural tube-like structures. After 30 days transplanted cells differentiate into mature neurons that express Neurofilaments and NeuN and also extend long axons into the host’s spinal cord. In addition, the transplanted cells improved growth and regeneration of host axons that are normally refractory and unable to grow. In summary, we have established an experimental paradigm to study the mechanisms that allow NSPC from regenerative pre-metamorphic frogs to improve spinal cord regeneration in non-regenerative froglets.

## Materials and Methods

### Husbandry and Surgery of *Xenopus laevis*

Wild Type frogs were obtained from Nasco (Fort Atkinson, WI, USA). Adult transgenic animals Xla.Tg(CAG:Venus)^Ueno^ (NXR_0.0006) and Xla.Tg(tubb2b:GFP)^NXR^ (NXR_0.0035) were obtained from the National Xenopus Resource (NXR Marine Biological Laboratory, Woodshole, MA, USA; Pearl et al., 2012). Adults were subjected to natural mating and raised until they achieved Nieuwkoop-Faber stage 50 (NF stage 50; R stage) or NF stage 66 (NR-stages; Nieuwkoop and Faber, 1994). For all surgical procedures, animals were anesthetized by incubation in 0.02% MS222, also known as Tricaine (Ethyl 3-aminobenzoate methanesulfonate, Sigma-Aldrich, A5040). 2 min for tadpoles and 10 min for froglets. Spinal cord resection and postoperative manipulation were executed as previously described (Edwards-Faret et al., 2017). Briefly, anesthetized froglets were moved to a gauze on top of a 10 cm petri dish, the dorsal skin was opened with a longitudinal incision above the spinal cord using microscissors, followed by careful lift of the dorsal muscles to expose the vertebrae. A dorsal laminectomy was performed between the 5th and 6th vertebrae using forceps followed by a complete resection of 1 mm of the spinal cord. All animal procedures were approved by the Committee on Bioethics and Biosafety from the Faculty of Biological Sciences, Pontificia Universidad Católica de Chile. Dr. Manuel Santos Alcántara, Dr. Waldo Cerpa Nebott, Dr. Luis Larrondo Castro, Dr. Ricardo Moreno Mauro, Sra. Micaela Ricca, Prof. Nicolás Rozbaczylo Narváez. Number: CBB-122/2013.

### Transplantation Experiments

Transplantation experiments were based on two previous works (Lu et al., 2012; Lin et al., 2013). Spinal cords were dissected from anesthetized animals and dissociated enzymatically by incubation in StemPro Accutase (Gibco, A1110501) and vortexing at room temperature for 1 h (NF stage 50) or 2 h (NF stage 66). Then, the single cell suspension was incubated with Trypan Blue (Sigma-Aldrich, T8154) and living cells counted using a hemocytometer. Cells were centrifuged at 200 RCF for 5 min, resuspended in L-15 Medium (Sigma-Aldrich, L4386) and supplemented with 0.5 mg/mL BSA (Sigma-Aldrich, A7906), 10 ng/mL FGF2 (R&D System 3139-FB-025/CF), 10 ng/mL EGF (R&D System 2028-EG-200), and 10 ng/mL BDNF (R&D System 248-BD-025/CF). This cocktail has the purpose of improving survival of transplanted cells. Then, the cell suspension was mixed with a 1:1 volume of 100 mg/mL of Fibrinogen (Sigma-Aldrich, F6755) and 100 U/mL Thrombin (Sigma-Aldrich, T5772), these proteins will form a clot and retain the cells avoiding its dispersion when transplanted. A suspension containing 100,000 cells were transplanted into the spinal cord of NF stage 66 animals resected as described above. Control animals received the same cocktail of GF together with fibrinogen and thrombin but without cells. For axon labeling at 1 week before the end of the experiment, reticulospinal and vestibulospinal tracts were anterogradely labeled by injection of 60 nl of 15% biotinylated dextran amine (BDA; MW 10,000, Thermo-Fisher, D1956) into the right and left medial-lateral hindbrain (Nieuwenhuys et al., 1998).

### Immunofluorescence and Immunohistochemistry

Tissue processing and immunofluorescence were performed as described (Muñoz et al., 2015) with minor modifications. Briefly, animals were anesthetized in 0.2% MS222 per 10 min, they were fixed by transcardial perfusion with 4% PFA in 0.8× calcium and magnesium-free Dulbecco’s PBS (CMF-PBS). The spinal cord containing vertebrae was dissected and then decalcified incubating the cords in 0.5 M EDTA in CMF-PBS (pH 7.8, 4°C) during 24 h at 4°C in a nutating mixer (Harms et al., 2002). Tissues were cryoprotected with increasing concentration of sucrose (from 10% to 30%) and OCT (Tissue-Tek 25608-930). Cryosections of 10 μm were obtained and were permeabilized with 0.2% Triton X-100 in CMF-PBS (PBST) at room temperature for 10 min, blocked with 10% inactivated goat serum diluted in PBST for 30 min, incubated with primary antibodies overnight at 4°C, secondary antibodies for 2 h at room temperature and stained with Hoechst 33342 (Thermo Fisher Scientific H1399). Antibodies used were mouse mAb anti-Sox2 (1:200, Cell Signaling Technology, L1D6A2), rabbit pAb anti-GFP (1:200, Abcam, ab6556), rabbit pAb anti-Neurofilament-200 (1:1000, Sigma-Aldrich, N4142), NeuN (1:1000 SDIX, Custom made, see below) and Alexa Fluor 488 or 555 (1:500; Invitrogen, Carlsbad, CA, USA), donkey pAb anti-Rabbit conjugated HRP (1:500, Invitrogen, Carlsbad, CA, USA, SA1-200) as secondary antibodies. Regarding the specificity of the primary antibodies, it is important to mention that the peptide used for the preparation of the anti-Sox2 antibody is conserved in Sox3; therefore, is not possible to rule out that this antibody also recognizes Sox3 (Muñoz et al., 2015). The antibody against Neurofilament-200 has been previously used in *X. laevis* (Lin et al., 2007). We used Streptavidin Alexa Fluor 488 conjugate for BDA detection (1:200, Invitrogen, Carlsbad, CA, USA, S11223). For immunohistochemistry, we used a pretreatment with H_2_O_2_ 1% in PBS for 15 min at room temperature. Then blocked and incubated with antibodies as mentioned before, the detection was made with DAB Peroxidase (HRP) Substrate Kit (with Nickel), 3,3′-diaminobenzidine for 10 min at room temperature (Vector Laboratory, SK4100).

### Preparation of Antibodies Against NeuN

A specific peptide for *Xenopus laevis* NeuN was designed using the fox3-derived sequence (BC088942.1) as a template (Kim et al., 2009). The sequence used as an epitope was NH2-MAQPYSTTQYPQPPQNGLPAEYASQHPLPTPDYSGQTTVSEHALTLYTA-GHSHGEPQGNEVSTQSVTGTQTLTTDEVSQTDSSQQLQCPESTDKQQPKRLH-ami-de, corresponding to the first 100 amino acids of the fox3 sequence. This peptide was used by Strategic Diagnostics Inc. (SDIX) to prepare a polyclonal antibody in two unpooled rabbits. The antibody was affinity purified and titrated over the recombinant peptide. The antibody only recognizes two bands of an apparent molecular weight (MW) of 47 kDa and 51 kDa (see Supplementary Figure S1), that is very similar to the MW previously described for NeuN (Dent et al., 2010), suggesting that the antibody is very specific for NeuN.

### Image Analysis

The quantifications were made using Fiji software (Schindelin et al., 2012). For area analysis we measured the area covered by Sox2/3 positive cells plus their ependymal lumen, after quantifying all the neural tube-like structures in one section we measured the total transplanted area, this was made for four cryosections from three different transplanted animals in each day. For axon regeneration, we measured the linear distance from the tip of an axon to the rostral stump in at least five non-consecutive (spaced by at least 120 μm) cryosections per animal, representative of the dorsoventral axis and relative to total axon quantified.

### Statistical Analysis

Statistical analysis was performed with Graphpad Prism software. One-way analysis of variance (ANOVA) followed by Bonferroni *post hoc* test were used to analyze the area of Sox2/3^+^ cells inside the graft over time.

## Results

### Spinal Cord Cells from Regenerative Larvae form Neural Tube-Like Structures after Transplantation into Non-regenerative Froglets

**Figure 1:**
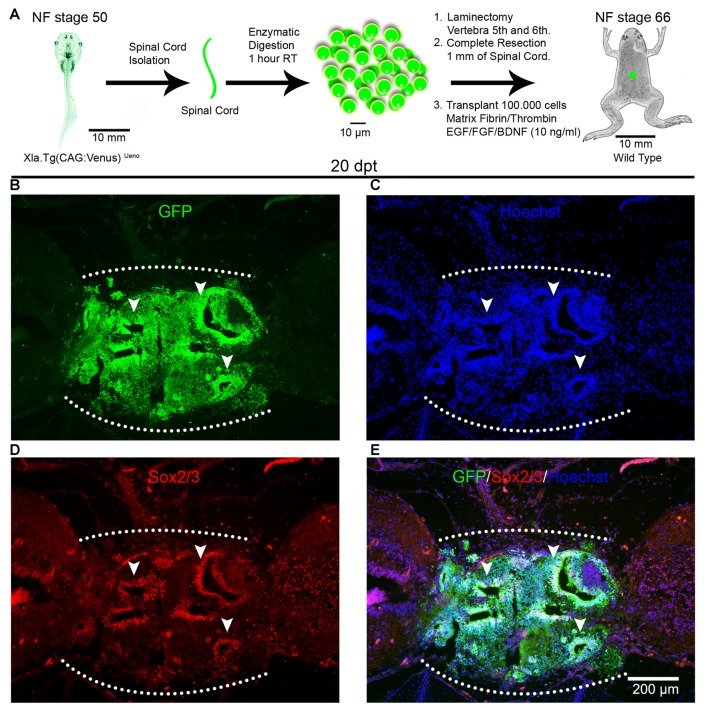
Spinal cord cells from regenerative larvae form rosette-like structures after transplantation into non-regenerative froglets. **(A)** Diagram of a heterochronic transplantation experiment using the transgenic line Xla.Tg(CAG:Venus)^Ueno^ as donor. **(B–D)** Sagittal sections of spinal cords from Nieuwkoop and Faber (NF) stage 66 froglets at 20 days post transplantation (dpt) stained with **(B)** α-GFP antibodies (green), **(C)** Hoechst (blue), **(D)** α-Sox2 antibodies and **(E)** a merge panel. Arrowheads point to rosette-like structures formed by donor cells. Rostral is left and caudal is right. The graft is demarcated with a white dotted line. Similar results were obtained in 10–12 independent experiments. Scale bar 200 μm.

To evaluate the ability of spinal cord cells from animals at R- and NR-stages to survive and engraft in the environment of a non-regenerative injured spinal cord, we developed a transplantation procedure based on protocols previously used for spinal cord transplantations in rats (Lu et al., 2012) and limb regeneration experiments in *X. laevis* (Lin et al., 2013). For heterochronic experiments, corresponding to transplantation experiments using donor and host animals from different stages, we used as donors animals at NF stage 50 (R-stage) from the transgenic line Xla.Tg(CAG:Venus)^Ueno^ that ubiquitously express the Venus reporter gene under the control of the CAG promoter (cytomegalovirus enhancer fused to the chicken beta-actin promoter) and wild type juvenile froglets at NF stage 66 (NR-stage) as host (Figure 1A). Spinal cords from donor animals were isolated, enzymatically dissociated and the cells supplemented with a cocktail of GF including FGF2, EGF and BDNF and mixed with a matrix of Fibrinogen and Thrombin and then transplanted into the injury site of host animals immediately after spinal cord resection. At 20 days post transplantation (dpt) GFP^+^ cells from the donor were engrafted in the injury site of host froglets and 93% of the cells in the graft come from the donor’s spinal cord and no GFP^+^ cells were found outside the transplantation site (Figures 1B,C). Interestingly, most of the GFP^+^ cells self-organize in rosettes structures that mimic the organization of the neural tube and most of the cells present in these structures come from the donor (Figures 1B,C, see arrowheads).

Although from the experiment depicted above it was clear that most of the cells in the graft come from the donor spinal cords we performed an experiment to determine if cells from the host were recruited to the injury site by the presence of the GF cocktail and the fibrin/thrombin matrix. For this similar heterochronic transplantation experiments were performed but in this case, we also included a control experiment were the host froglets only received the matrix and the GF cocktail without cells. Hematoxylin and eosin staining showed that at 10 dpt host animals that received donor cells already showed the formation of rosette structures and cells engrafted in the transplantation site, but only the matrix was observed in the control experiments (see Supplementary Figures S2A–D). At 40 dpt, we observed, even more, cells engrafted in the injury site of animals that received donor cells, but no cells were observed in the control experiments and also at this day the matrix was already degraded and reabsorbed (see Supplementary Figures S3A–D).

**Figure 2:**
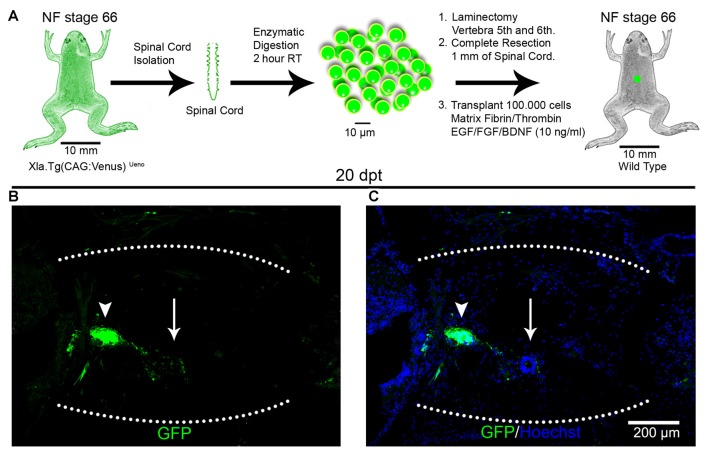
Transplantation of spinal cord cells from non-regenerative froglets. **(A)** Diagram of a homochronic transplantation experiment using the transgenic line Xla.Tg(CAG:Venus)^Ueno^ as donor. **(B,C)** Sagittal sections of spinal cords from NF stage 66 animals at 20 dpt stained with **(B)** α-GFP antibodies (green) and **(C)** Hoechst (blue). The graft is demarcated with a white dotted line. Scale bar 200 μm.

To test if cell survival, engraftment and rosette formation were specific for cells from the spinal cords obtained from R-stage larvae, we performed homochronic experiments, corresponding to transplantation experiments using donors and host animals from the same stages. For this, we used NR-stage froglets from the transgenic line Xla.Tg(CAG:Venus)^Ueno^ as donors and wild type animals at the same stage as hosts (Figure 2A). Spinal cords from the donor animals were isolated, cells dissociated and transplanted into NR-stage froglets using the same conditions described above. Despite the fact that the same amount of living cells were transplanted, at 20 dpt only a few GFP^+^ cells were found in the injury site forming very small rosette structures (Figures 2B,C; arrowheads). We also observed rosettes formed by GFP negative cells (Figures 2B,C; arrows). These results demonstrate that the spinal cords from R-stage larvae contain cells that are able to survive and form rosette structures in a non-regenerative host and that cells with the ability to survive after transplantation were no longer present or present at very low levels in the spinal cord of non-regenerative animals.

**Figure 3:**
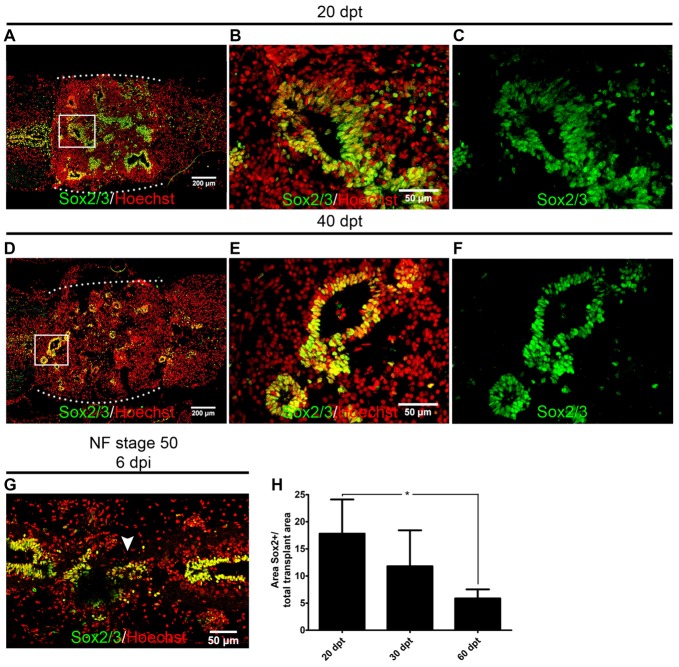
Sox2/3^+^ cells from regenerative stage (R-stage) spinal cords form neural tube-like structures when transplanted into non-regenerative stage (NR-stage) froglets. **(A–F)** Longitudinal sections of spinal cords from NF stage 66 froglets stained with α-Sox2 antibodies (green) and Hoechst (red) at **(A–D)** 20 dpt and **(D–F)** 40 dpt. Panels **(B,C,E,F)** are magnifications of the boxed areas in panels **(A,D)**, respectively. The white dotted line indicated the grafted cells. **(G)** Longitudinal section of the spinal cord from R-stage animals at 6 days post injury (dpi) stained with α-Sox2 antibodies (green) and Hoechst (red). Arrowhead indicates the formation of a neural tube-like structure in the injury site. Rostral is left and caudal is right. **(H)** Quantification of the area covered with neural tube-like structures at different days after transplantation. Error bars are standard deviations; *n* = 3 per day; one-way analysis of variance (ANOVA) test analysis showed significant differences between 20 dpt and 60 dpt. *p < 0.05. Scale bar 200 μm and 50 μm.

We have demonstrated previously that cells expressing Sox2/3 are very abundant in the central canal of the spinal cord at R-stages and only a few Sox2/3^+^ cells were present in NR-stages (Gaete et al., 2012; Muñoz et al., 2015). In addition, we have demonstrated that SCI in R-stages induces massive proliferation of Sox2/3^+^ cells and this proliferation is necessary for spinal cord regeneration (Muñoz et al., 2015). Because of this, we decided to evaluate the possibility that Sox2/3^+^ cells are involved in the formation of rosette structures after heterochronic transplantation. First, we check if the transgenic line used for transplantation experiments express GFP in the spinal cord, and we found that most Sox2/3^+^ cells in the ependymal layer of R-stage larvae expressed GFP (see Supplementary Figure S4). Then we performed immunofluorescence against Sox2/3 in animals after heterochronic transplantation and found that most of the GFP^+^ cells present in the rosette structures expressed Sox2/3 (Figures 1B,D,E). Quantification of these structures showed that at 20 dpt rosette structures containing Sox2/3^+^ cells occupied approximately 17% of the graft and only 6% at 60 dpt (Figures 3A,D,H). Of note, each rosette observed have different level of Sox2/3 expression along its long axis (Figures 3B,C,E,F) that is very similar to the dorsal to ventral gradient expression found in the spinal cord of R-stage animals (Edwards-Faret et al., under revision). These rosettes structures were very reminiscent of the structures formed by Sox2/3^+^ cells in the injury site at 6 days after SCI (dpi) in R-stage larvae (Figure 3G, arrowhead). Because of these similarities, we conclude that the rosettes correspond to neural tube-like structures formed by Sox2/3^+^ cells from the donor.

In summary, we have set-up cell transplantation experiments into the injury site after spinal cord transection in *X. laevis* froglets. We found that in heterochronic transplantation donor cells from R-stages are able to engraft in the injured spinal cord of froglets and self-organize forming neural tube-like structures mainly containing donor cells expressing Sox2/3 and cells from the host were not able to populate the graft. In homochronic transplantation experiments, spinal cords from NR-stages were used as donors and very few cells survived and engrafted.

### Spinal Cord Cells from Regenerative Animals Differentiate into Neurons and Extend Axons after Transplantation into the Non-regenerative Spinal Cord

**Figure 4:**
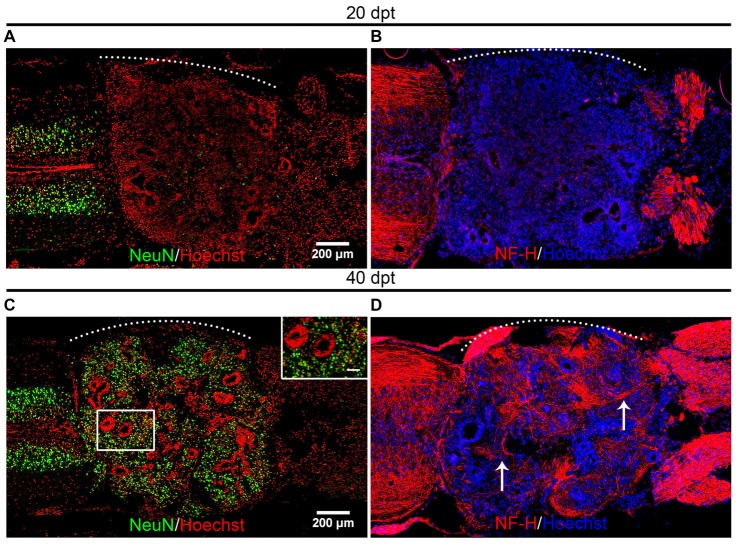
Mature neurons are present in the graft. **(A–D)** Longitudinal sections of the spinal cord of NR-stage froglets that received cells from R-stage animals and were stained for **(A,C)** NeuN or **(B,D)** Neurofilament-Heavy chain (NF-H) at **(A,B)** 20, **(C,D)** and 40 dpt. Inset in panel **(C)** showed nuclear localization of NeuN. Arrows indicate axon staining. The white dotted line indicates the transplantation site. Rostral is left and caudal right. Scale bar 200 μm and 50 μm for inset in panel **(C)**.

Based on the finding that most of the transplanted cells are Sox2^+^ cells and that we have previously demonstrated that these cells can make new neurons after SCI in the context of the regenerative animals (Muñoz et al., 2015) we decided to evaluate if the transplanted cells were able to differentiate and form new neurons in the non-regenerative host. For these, we used the experimental paradigm of heterochronic transplantation but non-transgenic animals were used as donors. To evaluate the differentiation state of the transplanted cells we performed immunofluorescence against NeuN, a marker expressed in mature neurons, and also for Neurofilament-Heavy chain (NF-H) for axon identification. At 20 dpt very few cells positive for NeuN or NF-H were found in the graft (Figures 4A,B). On the contrary, 40 dpt a high number of cells expressing NeuN in the nuclei and NF-H positive axons were found in the graft (Figures 4C,D, see arrows), indicating that neural differentiation requires at least 4 weeks.

**Figure 5:**
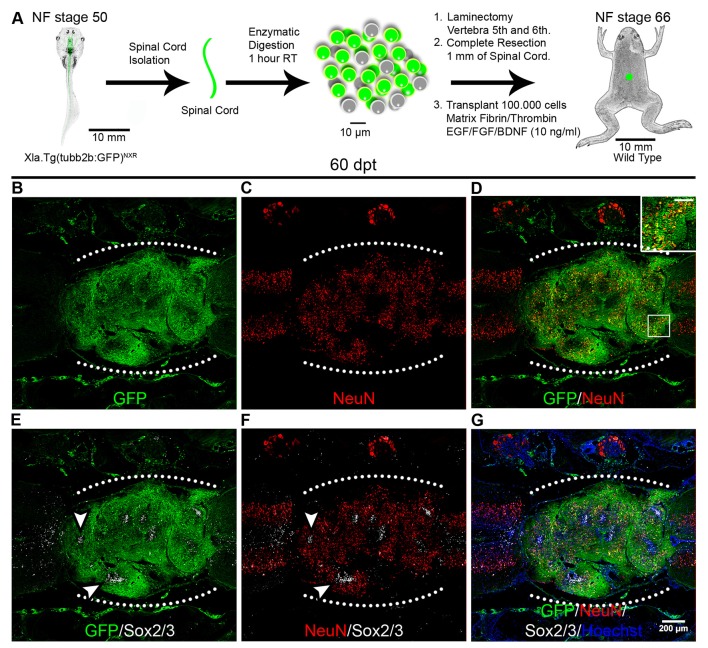
Donor cells differentiate into neurons after transplantation. **(A)** Diagram of a heterochronic transplantation experiment using the transgenic line Xla.Tg(tubb2b:GFP)^NXR^ as donor. **(B–G)** Longitudinal sections of the spinal cord of NR-stage froglets that received cells from R-stage animals and were stained for GFP, NeuN, Sox2 and Hoechst. Inset in panel **(D)** showed co-localization of GFP and NeuN, scale bar 50 μm. Arrowheads indicate GFP negative regions expressing Sox2/3. The white dotted line indicated the transplantation site. Rostral is left and caudal right. Similar results were obtained in two independent experiments. Scale bar 200 μm.

To determine if the mature neurons and axons present in the graft are derived from the donor or the host we performed heterochronic transplantation using as donors spinal cord cells from R-stage larvae from the transgenic line Xla.Tg(tubb2b:GFP)^NXR^ that express GFP under the control of the N-tubulin promoter (tubulin beta 2B class IIb promoter) allowing the expression of GFP in all neurons (Figure 5A). At 60 dpt expression of GFP was detected in almost the entire graft in some nuclei but mainly in axonal staining (Figure 5B). Neurons expressing NeuN in the graft were positive for GFP indicating that were derived from the donor cells (Figures 5C,D; see inset). Interestingly, the domains of the graft that were GFP negative correspond to cells that express Sox2, in agreement with the expression pattern of the donor transgenic line that only expresses GFP in neurons but not in neural progenitors, and this Sox2/3^+^ cells were surrounded by cells expressing NeuN and GFP^+^ axons (Figures 5E–G; see arrowheads).

**Figure 6:**
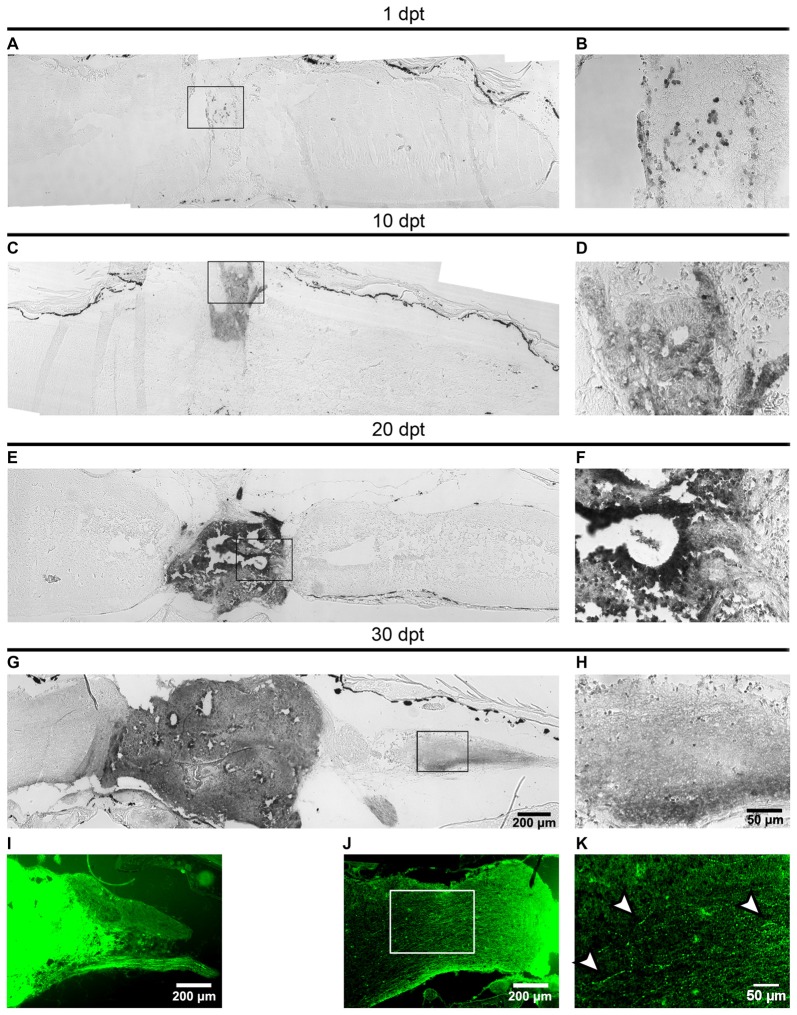
Axons from the transplanted cells growth into the host spinal cord. **(A–I)** Sagittal sections of host spinal cords that received cells from R-stage animals and were stained with immunohistochemistry against the Venus protein at 1, 10, 20 and 30 dpt. Panels **(B,D,F,H)** are magnifications of the boxed areas depicted in panels **(A,C,E,G)**, respectively. Axons extend 650 ± 350 μm into the caudal region of the host spinal cord. **(I–K)** Sagittal sections showing the GFP signal in the host spinal cord 30 dpt. Arrowheads in panel **(K)** indicate possible varicosities present in axons growing into the host animal. Axons extend 800 ± 100 μm into the rostral region of the host spinal cord. Rostral is left and caudal right. Similar results were obtained in 2–6 independent experiments Scale bar 200 μm and 50 μm.

One dogma in CNS regeneration is that axons are not able to regenerate in the non-permissive environment present in the adult CNS (Richardson et al., 1980; Xie and Zheng, 2008). Recent experiments showed that NSC transplanted into an injured spinal cord rat, were able to differentiate into neurons, and a massive amount of axons was able to grow in the non-permissive environment (Lu et al., 2012). To evaluate if the mature neurons formed from transplanted cells can grow axons into the non-regenerative hosts we used the Xla.Tg(CAG:Venus)^Ueno^ transgenic animals as donors followed by immunohistochemistry against Venus at 1, 10, 20 and 30 dpt. At 1 dpt few GFP^+^ cells were found in the matrix grafted into the injury site (Figures 6A,B). At 10 dpt more GFP^+^ cells were present in the injury site and neural tube-like structures were already detected (Figures 6C,D). At 20 dpt the graft contains many donor cells organized in neural tube-like structures that fill the injury gap suggesting that extensive proliferation of donor cells has occurred during this time period (Figures 6E,F).

At 30 dpt the clone of transplanted cells is even larger and more importantly GFP^+^ axons extend 650 ± 350 μm into the caudal region of the host the spinal cord and GFP^+^ peripheral motor nerves were observed (Figures 6H,I). Observation of GFP fluorescence also allows the detection of axons extending 800 ± 100 μm into the rostral region of the host spinal cord (Figure 6J) at higher magnification structures similar to varicosities were observed (Figure 6K, see arrowheads).

In summary, we provide evidence that cells from regenerative animals, most probably Sox2/3^+^ cells, are able to differentiate into mature neurons in the non-regenerative host. During the first 20 dpt, most transplanted cells remain undifferentiated and almost no expression of NeuN and NF-H was detected in the host, concomitant with the decrease in the number of Sox2/3 cells that occurs during this time period (Figure 3H). At later days we observed an increase in the appearance of mature neurons (NeuN^+^; NF-H) and axons derived from the transplanted cells are able to grow into the host spinal cord. These results suggest that Sox2/3 cells present in the spinal cord of R-stage larvae are most probably NSPC that are able to survive, proliferate, self-organize and differentiate into neurons in the environment of the non-regenerative host.

### Spinal Cord Cells from Regenerative Animals Provides a Permissive Substrate for the Growth and Regeneration of Axons from Non-regenerative Animals

The absence of intrinsic programs and a non-permissive environment are the main reasons to explain why axon regeneration fails in mammals and other non-regenerative organisms. It has been reported previously that frogs lose the ability for axon regeneration around NF stage 60, implicating that NF stage 66 animals are not able to regenerate their axons (Beattie et al., 1990). To evaluate the ability of cells from R-stage spinal cords to promote axon growth and regeneration in non-regenerative froglets we carried out two experimental approaches.

**Figure 7:**
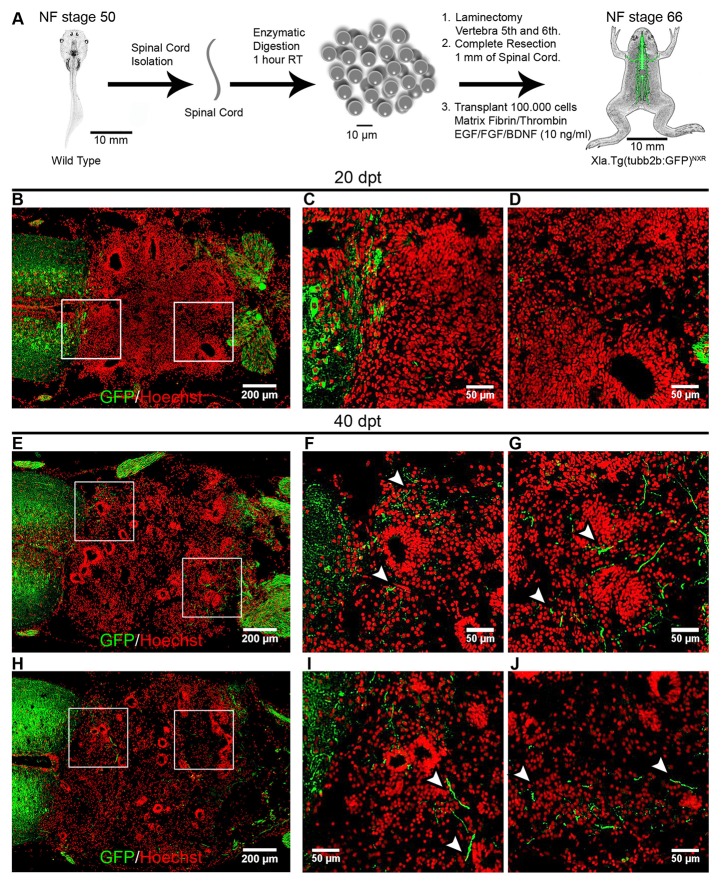
Transplanted cells from R-stages provide a permissive substrate that promotes axon growth. **(A)** Diagram of the transplantation procedure. **(B–J)** Longitudinal sections of transplanted NR-stage froglets were stained with α-GFP antibodies at 20 and 40 dpt. Arrowheads indicate GFP^+^ axons growing up to 200 ± 50 μm into the transplantation site. Rostral is left and caudal is right. Similar results were obtained in three independent experiments. Scale bar 200 μm and 50 μm.

First, we used wild type NF stage 50 animals as donors and for hosts we used NF stage 66 froglets from the transgenic line Xla.Tg(tubb2b:GFP)^NXR^ (Figure 7A). Immunofluorescence analysis against GFP were performed at 20 dpt and 40 dpt. Very few axons growing into the graft were observed at 20 dpt (Figures 7B–D). Importantly, at 40 dpt many GFP positive axons growing into the transplanted cells were detected and extend up to 200 ± 50 μm into the transplantation site (Figures 7E–J, arrowheads).

**Figure 8:**
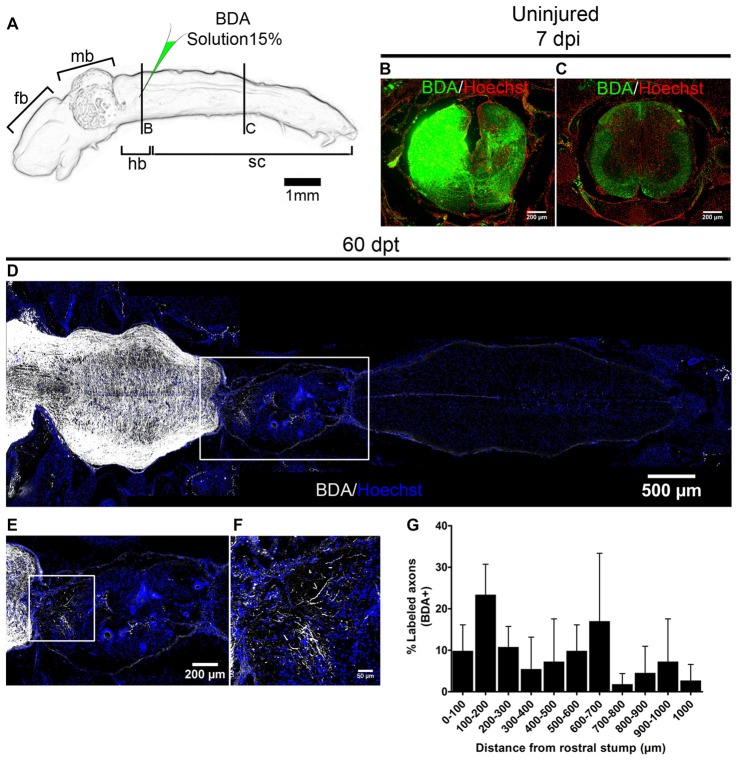
Transplanted cells from R-stages promote axon regeneration. **(A)** Drawing of the central nervous system (CNS) from NR-stage froglets showing the approximate site of biotinylated dextran amine (BDA) injection. **(B,C)** Transverse sections of animals injected on one side of the brainstem in a region that is **(B)** near or **(C)** 3 mm caudally to the injection site. **(D–F)** Longitudinal sections of transplanted NR-stage froglets, that received BDA injections as indicated in panel **(A)** and were stained for BDA (white) or nuclei (blue). Panel **(E)** is a magnification of the area depicted in panel **(D)** and panel **(F)** a magnification of the boxed area from panel **(E)**. Rostral is left and caudal is right. Scale bar 200 μm and 50 μm. **(G)** Quantification of regenerated axons across the transplant (*n* = 3). Error bars are standard deviations.

To evaluate if the transplanted cells can promote regeneration of injured axons we performed anterograde labeling (Tuszynski and Steward, 2012). Anurans do not have corticospinal tracts or tracts that descent directly from telencephalon into the spinal cord (Nieuwenhuys et al., 1998). But it is known that brainstem nuclei regenerate in R-stage animals (Beattie et al., 1990) and for that reason, we labeled descending tracts in NF stage 66 animals injecting BDA in one side of the hindbrain (Figure 8A). Seven days after injection we found the anterograde tracer mainly in the injected site (Figure 8B) and also in regions of the spinal cord that are 3 mm distal to the injected site indicating successful labeling of those tracts (Figure 8C). To test the ability of R-stage spinal cord cells to promote axon regeneration in non-regenerative froglets we transplanted spinal cord cells as depicted above and at 53 dpt both sides of the hindbrain were injected with BDA and animals fixed 1 week later and processed for immunofluorescence to detect BDA. We have found many labeled axons growing into the transplanted cells up to 1 mm from the rostral stump (Figures 8D–G). Qualitative analysis of one of transplanted animal showed partial movement in its right hindlimb (data not shown), although a quantitative test to measure functional recovery needs to be developed.

In summary, we provide evidence that spinal cord cells from regenerative animals provide a permissive substrate that promotes axons from the non-regenerative animals to grow and regenerate into the injury site.

## Discussion

*X. laevis* has been used extensively as a model system to study developmental biology and many transplantation experiments had been performed at embryonary stages. However, transplantation experiments in larvae and froglets are less common. One of the few experiments reported is the transplant of limb bud cells between regenerative and NR-stage (Lin et al., 2013). Here we adapted a protocol used in rats in order to study the properties of cells present in the spinal cord of R-stages of *X*. *laevis*. We found that some cells were able to survive and engraft under the injury conditions present in NR-stages even when transplanted immediately after the injury was performed. Something that is not observed in mammals was cells usually die when they are transplanted immediately after injury (Tetzlaff et al., 2011). Although, we have not checked how many cells die during the first days after transplantation, the fact that a few scattered cells are observed at 1 dpt but then at 10, 20 and 30 dpt (compare Figure 6A with Figures 6C,E,G) many more cells are engrafted indicates that the transplanted cells that survive are able to proliferate and fill the injury site. This observation of growth after a phase of cell death is in line with recent reports in transplantation experiments performed in rodents (Lu et al., 2017).

We have found that graft is formed by cells from the donor and no cells derived from the host are found in the graft. Donor cells in the graft can self-organize in neural tube-like structures formed by Sox2/3^+^ cells that are very reminiscent of structures formed in the injury site after spinal cord transection of R-stage animals. This type of rosette structures have been observed in the formation of different organs during development, including the formation of the neural tube and in the subventricular zone (Harding et al., 2014). In addition, the formation of rosette structures is a hallmark of *in vitro* neural differentiation of human Embryonic Stem Cell (hESC) and are dependent on the presence of FGF2 (Zhang et al., 2001). Interestingly, FGF is necessary for the formation of “glial-bridges” and subsequent axon regeneration in the spinal cord of Zebrafish (Goldshmit et al., 2012), These pieces of evidence suggest that we could be inducing rosette formation with the addition of FGF2, but could also be produced by a self-organization mechanism of the Sox2/3^+^ cells from the R-stage larvae.

The process of differentiation of the transplanted cells follows what occurs during normal development of the nervous system. During the first weeks, the transplanted cells proliferate with the concomitant increase in the size of the graft, cells organize in neural tube-like structures and express Sox2/3^+^ but no markers of neural differentiation were detected. Three to four weeks after transplantation a decrease in the Sox2/3^+^ neural tube-like structures was detected accompanied by the expression of markers such as NeuN and NF-H and the ability of axons from the graft to grow into the host spinal cord. All these results indicate that transplanted cells can proliferate and differentiate into mature neurons suggesting that the R-stage spinal cord contains NSPC. The temporality of differentiation is quite remarkable because coincidently it takes 1 month for a tadpole to metamorphose into a froglet, this agrees with the recent evidence that human NSC transplanted into rats spinal cord retain an intrinsic temporality for differentiation (Lu et al., 2017).

Regarding the ability of axons from the graft to grow into the host spinal cord, we found axons growing into the rostral and caudal spinal cord, but also into peripheral motor nerves, suggesting that some motor neurons are present in our transplanted tissue. However, we have not use any marker for motoneurons such as Islet-1 or the homeobox gene Hb9. These results are different compared with experiments performed in rats (Lu et al., 2012), where axons growing away from transplanted cells were detected already at 1 day after transplantation. However, both protocols are not identical, among other differences we can mention that: (i) here we only have used three growth factors (FGF, EGF, BDNF) and they have used a cocktail of 14 GF; (ii) we used a concentration of GF that is 1000 less than the one used in rats; and (iii) we have performed cell transplantation immediately after injury and cells were transplanted into rats spinal cord 2-weeks after injury emulating a chronic clinical condition.

Transplanted cells promote axons from non-regenerative froglets to grow and regenerate. Previous work showed that axons after NF stage 60 are refractory to regenerate after injury (Beattie et al., 1990; Gibbs et al., 2011). Our results agree with the recent evidence reported in rats, indicating that severed axons sense a pro-regenerative signal in transplanted cells particularly when they are homotypic (Kadoya et al., 2016). This evidence argues against the idea of lack of a regenerative program and proposes the idea of a pro-regenerative program that can be turned on by a permissive environment that in this case is the presence of transplanted cells from regenerative animals. With the experiments reported here, we are not able to identify the specific population of neurons that are regenerating and the cells from the donor that promote axon growth and regeneration. In other regenerative species, meningeal and glial cells seem to promote axon regeneration (Zukor et al., 2011; Goldshmit et al., 2012).

Here we have established an experimental paradigm to study the mechanisms that allow NSPC from regenerative frogs to improve spinal cord regeneration in non-regenerative animals. Although we have found positive effects of transplanted cells at the anatomical and histological level in the conditions tested no significant functional recovery has been detected (data not shown). Many aspects to improve the efficiency of these transplantation experiments remain open for further exploration. Although we did not include any immune suppression in our transplantation experiments, immune rejection could probably explain the possible cell death during the first days after transplantation. In limb transplantation experiments it has been demonstrated the importance of suppressing the froglet immune system to improve survival of cells coming from NF stage 53 donors (Lin et al., 2013) supporting the possibility that better results could be obtained if immune suppression is included. In addition, it is known that better results could be attained when cells are transplanted 1–2 weeks after transplantation and when other GF are added (Tetzlaff et al., 2011; Lu et al., 2012). In the future, testing new conditions to improve the effects of cell transplantation in anatomical and histological recovery can be correlated with functional improvements using kinematic analysis in non-regenerative froglets (Beyeler et al., 2008).

Transplantation of NSPC has been proposed as a potential therapy to improve recovery after SCI. However, there is limited knowledge and understanding of the cellular and molecular mechanism through which these cells improve CNS function and which are the best conditions to attain significant efficiency. The most common mechanisms include neuroprotection, immunomodulation, axon regeneration, neuronal relay formation and myelin regeneration (Assinck et al., 2017). We envision that cellular transplantation studies between regenerative and non-regenerative *Xenopus* stages could aid in understanding the mechanisms that allow NSPC to promote recovery after damage to the spinal cord and also to identify the conditions to improve the efficacy of these therapies.

## Author Contributions

EEM-O performed all the experiments. EEM-O and JL designed the experiments, analyzed the results and wrote the manuscript. RM prepared the α-NeuN antibody.

## Conflict of Interest Statement

The authors declare that the research was conducted in the absence of any commercial or financial relationships that could be construed as a potential conflict of interest.

## Abbreviations

CNS: Central Nervous System
dpt: days post-transplantation
hESC: human Embryonary Stem Cell
NF: Nieuwkoop and Faber
NR-stage: Non-Regenerative Stage
NSPC: Neural Stem and Precursor Cell
R-stage: Regenerative Stage
SCI: Spinal Cord Injury.
