# Supplementary figures and images for: Spinal Cord Cells from Pre-metamorphic Stages Differentiate into Neurons and Promote Axon Growth and Regeneration after Transplantation into the Injured Spinal Cord of Non-regenerative Xenopus laevis Froglets
Source: Front Cell Neurosci. 2017 Dec 13;11:398. doi: 10.3389/fncel.2017.00398 (PMC5733487; doi:10.3389/fncel.2017.00398)

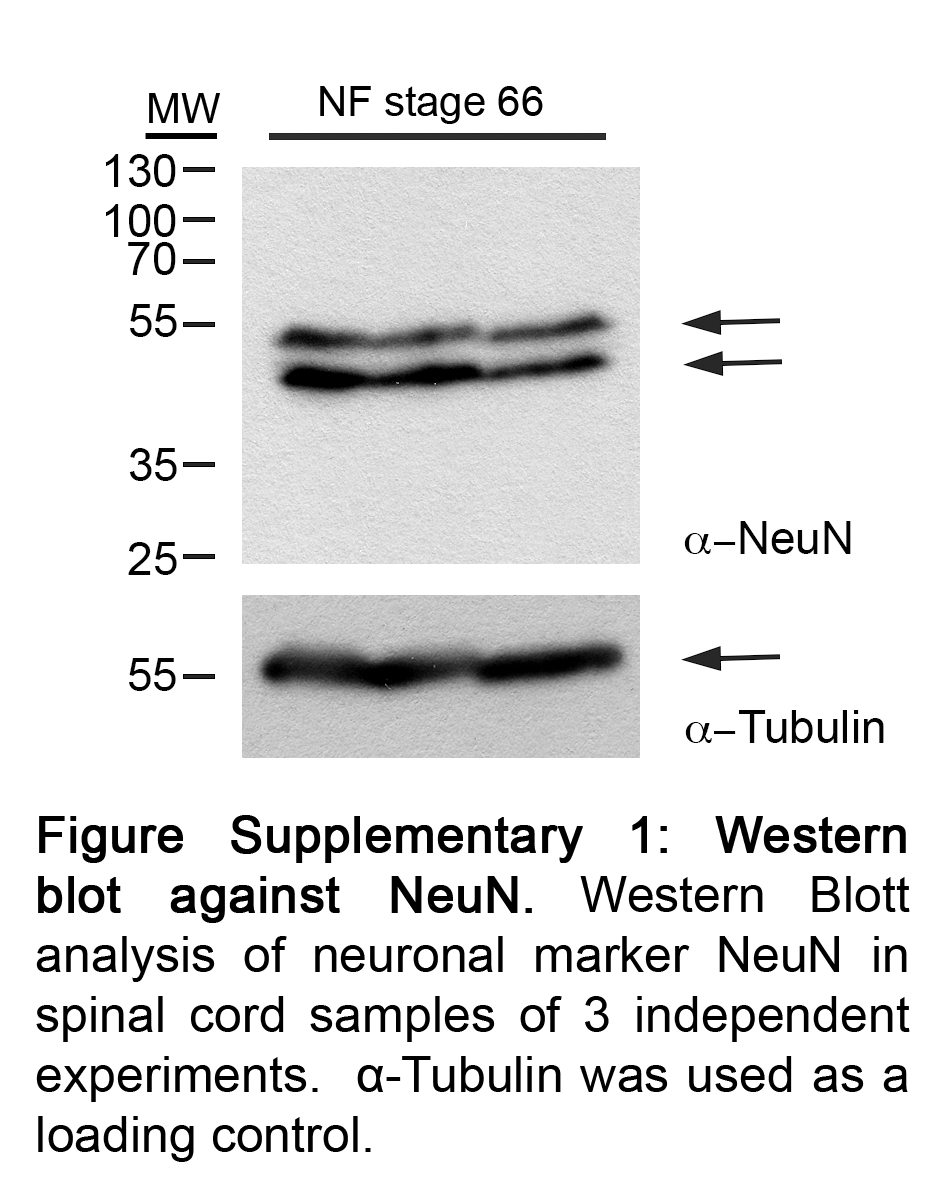

Supplement: Supplementary file 1 [file Image_1.tif]

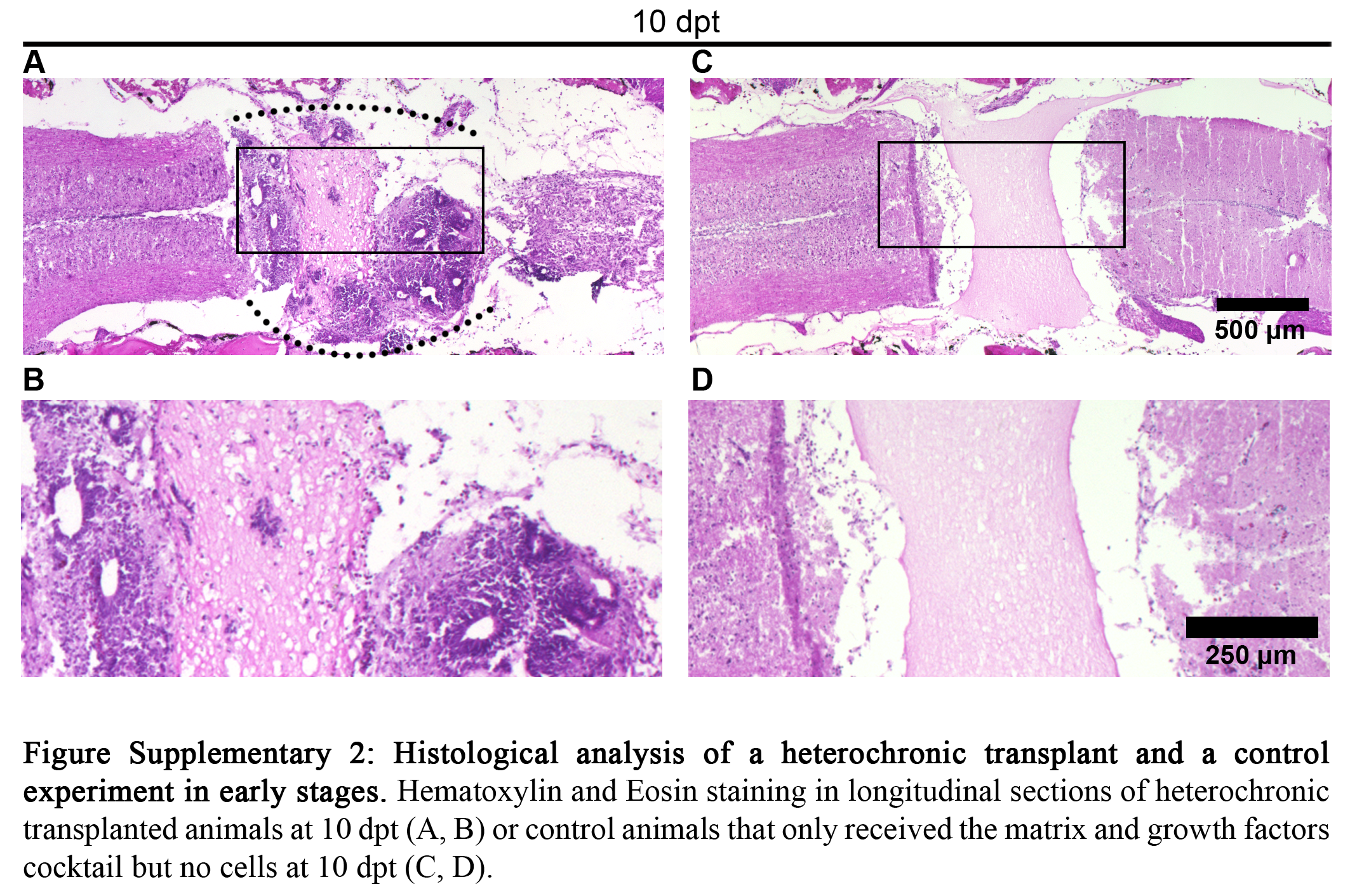

Supplement: Supplementary file 2 [file Image_2.tif]

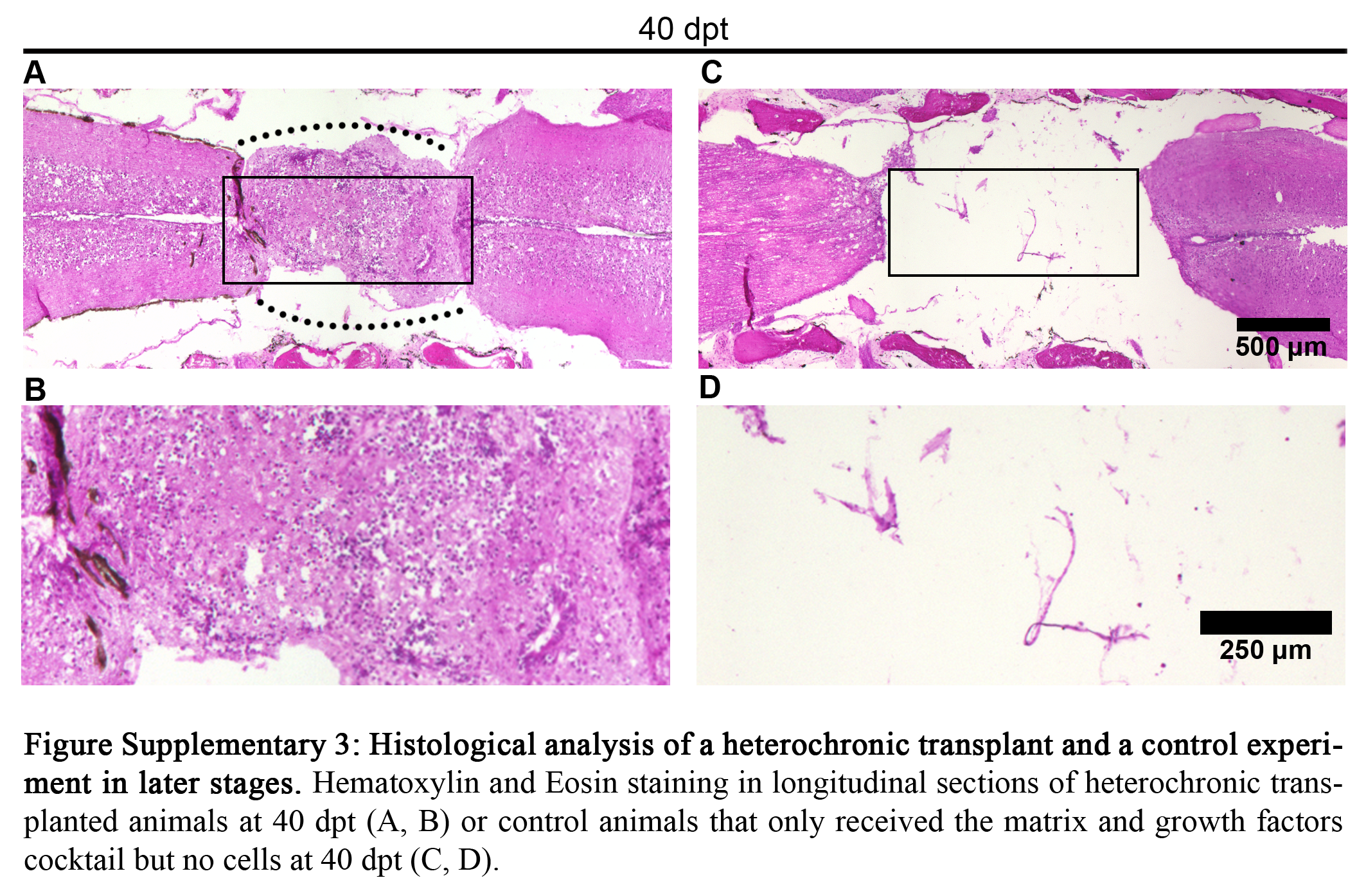

Supplement: Supplementary file 3 [file Image_3.tif]

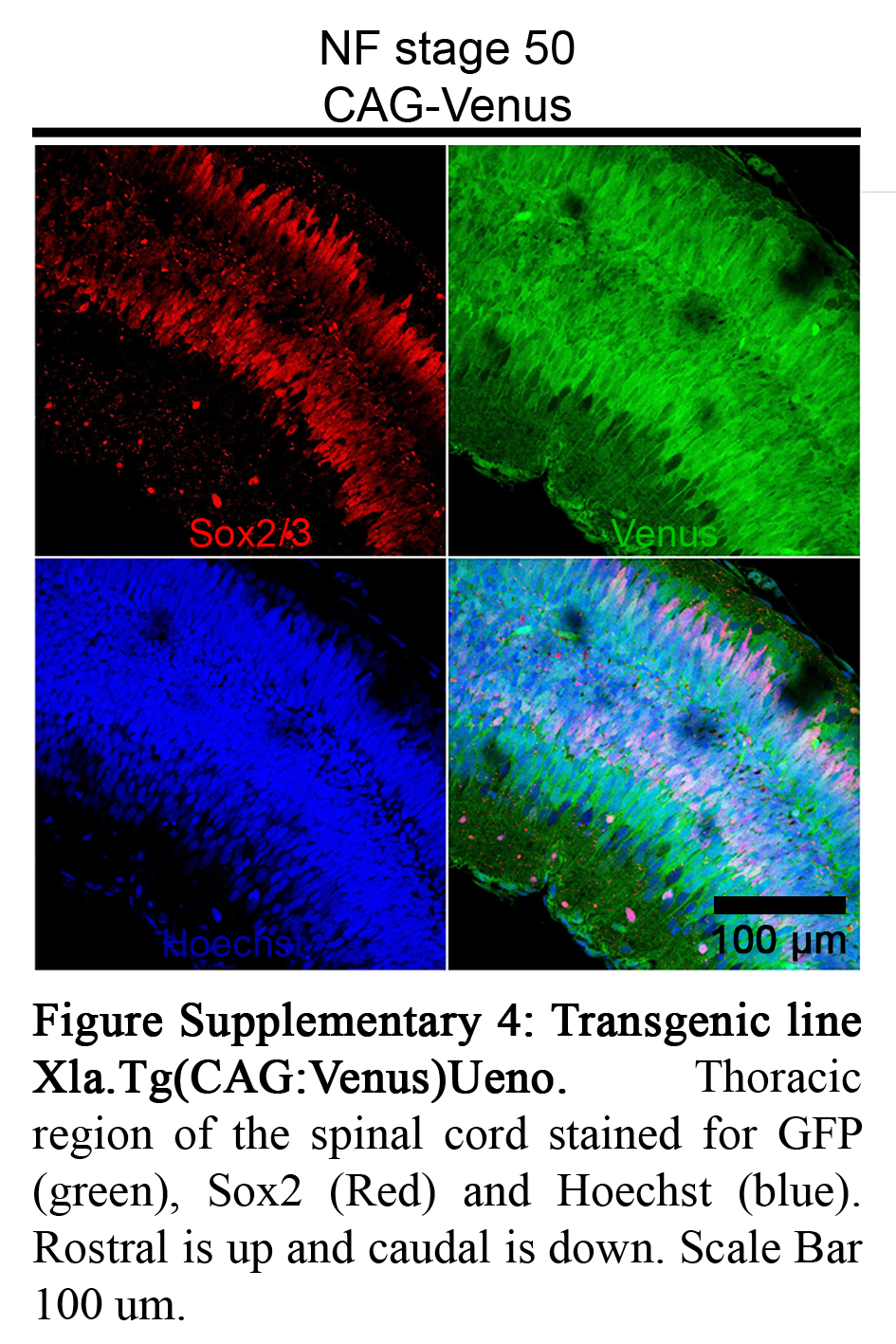

Supplement: Supplementary file 4 [file Image_4.tif]
